# Serum Magnesium Concentrations in the Canadian Population and Associations with Diabetes, Glycemic Regulation, and Insulin Resistance

**DOI:** 10.3390/nu9030296

**Published:** 2017-03-17

**Authors:** Jesse Bertinato, Kuan Chiao Wang, Stephen Hayward

**Affiliations:** 1 Nutrition Research Division, Food Directorate, Health Products and Food Branch, Health Canada, Sir Frederick G. Banting Research Centre, 251 Sir Frederick Banting Driveway, Ottawa, ON K1A 0K9, Canada; 2 Department of Biochemistry, Microbiology and Immunology, University of Ottawa, Ottawa, ON K1H 8M5, Canada; 3 Bureau of Food Surveillance and Science Integration, Food Directorate, Health Products and Food Branch, Health Canada, Ottawa, ON K1A 0K9, Canada; kuan.chiao.wang@hc-sc.gc.ca (K.C.W.); stephen.hayward@hc-sc.gc.ca (S.H.)

**Keywords:** Canada, diabetes, glycemic regulation, homeostatic model assessment of insulin resistance, serum magnesium concentration

## Abstract

Total serum magnesium (Mg) concentration (SMC) is commonly used to assess Mg status. This study reports current SMCs of Canadians and their associations with demographic factors, diabetes, and measures of glycemic control and insulin resistance using results from the Canadian Health Measures Survey cycle 3 (2012–2013). Associations were examined in adults aged 20–79 years using linear mixed models. Mean SMCs and percentile distributions for 11 sex-age groups between 3 and 79 years (*n* = 5561) are reported. SMCs were normally distributed and differences (*p* < 0.05) among sex and age groups were small. Between 9.5% and 16.6% of adult sex-age groups had a SMC below the lower cut-off of a population-based reference interval (0.75–0.955 mmol·L^−1^) established in the United States population as part of the NHANES I conducted in 1971–1974. Having diabetes was associated with 0.04 to 0.07 mmol·L^−1^ lower SMC compared to not having diabetes in the various models. Body mass index, glycated hemoglobin, serum glucose and insulin concentrations, and homeostatic model assessment of insulin resistance were negatively associated with SMC. This is the first study to report SMCs in a nationally representative sample of the Canadian population. A substantial proportion of Canadians are hypomagnesaemic in relation to a population-based reference interval, and SMC was negatively associated with diabetes and indices of glycemic control and insulin resistance.

## 1. Introduction

Magnesium (Mg) is a mineral nutrient that functions as a catalytic co-factor and structural component of enzymes and plays an important role as a calcium antagonist [1]. Mg is essential for many biological processes including the synthesis of organic molecules, cell proliferation, energy production, muscle contraction and relaxation, bone development, mineral metabolism, and glucose homeostasis [1,2,3,4]. In North America, Mg intakes fall short of dietary recommendations for a large segment of the population [5,6,7]. However, the extent of Mg deficiency in the general population and related health risks are unclear because of the uncertainty regarding Mg intakes needed for optimal health. This is underscored by the large differences in recommended intakes for Mg established by different scientific bodies [8,9,10]. In addition, current information on Mg status of the North American population is lacking.

Total serum Mg concentration is the most widely used nutritional biomarker for assessing Mg status [1,11,12]. A recent meta-analysis of randomized controlled trials showed that serum (or plasma) Mg concentrations were significantly increased by oral Mg supplementation in a dose- and time-dependent manner [13]. Notably, little or no change in serum Mg was observed with higher baseline circulating Mg concentrations. Together, these results suggest that serum Mg concentrations can provide meaningful information on Mg status.

Information on serum Mg concentrations in the Canadian or United States populations from nationally-representative health surveys is limited. The last national estimates were based on data collected over 40 years ago in the first National Health and Nutrition Examination Survey (NHANES I) conducted in the United States between 1971 and 1974 [14]. These estimates were used to establish a population-based reference interval for adults of 0.75–0.955 mmol·L^−1^. Based on this reference interval, a serum Mg concentration below 0.75 mmol·L^−1^ is usually defined as hypomagnesaemia (low serum Mg concentration).

Low Mg intakes and/or serum Mg concentrations have been associated with a number of diseases and health conditions including hypertension [15,16], sudden cardiac death [17,18], reduced bone mineral density [19], cardiovascular disease events [20,21], and colorectal cancer [22,23]. Poor Mg status may also impair growth of lean body mass [24] and decrease physical performance [25]. Diabetes (both type 1 and type 2) is the most common metabolic disorder associated with Mg deficiency [26,27,28,29,30], with reported incidence rates of hypomagnesaemia in diabetics as high as 13.5%–47.7% [27]. Multiple factors likely contribute to the hypomagnesaemia, including increased Mg loss through excretion by the kidneys. The higher renal Mg excretion is caused by reduced tubular Mg reabsorption resulting from glucose-induced osmotic diuresis and possibly insulin resistance [27,31]. Serum Mg has been negatively associated with fasting glucose and insulin concentrations and glycated hemoglobin (HbA_1c_), a measure of long-term glycemic control [32,33,34]. Negative associations have also been reported with indirect indices of insulin resistance including quantitative insulin sensitivity check index (QUICKI), homeostatic model assessment of insulin resistance (HOMA-IR), and McAuley’s index [32,35].

Nationally-representative data are preferred over non-national data for the development of nutrition policies and regulations. National estimates of serum Mg concentrations have never been reported for Canadians. The primary objective of this study was to report current national estimates of serum Mg concentrations for the Canadian population for ages 3–79 years using results from the Canadian Health Measures Survey (CHMS) cycle 3 conducted between 2012 and 2013. In Canada, the high prevalence of obesity [36] and co-morbidities such as insulin resistance and diabetes [37] may have a negative effect on the Mg status of the population. Thus, secondary analyses were performed to examine population-level associations between serum Mg concentrations and demographic factors, diabetes, and measures of glycemic regulation and insulin resistance in adults in order to add to our understanding of subpopulations at increased risk for Mg deficiency.

## 2. Materials and Methods

### 2.1. Survey Design

The CHMS is a cross-sectional, population-based survey that collects health information through a household interview and direct physical measures. The CHMS used a multi-stage sampling design. For cycle 3 of the survey, anthropometric measurements and biological samples were collected in a mobile examination centre (MEC) over 2 years from January 2012 to December 2013. Samples were collected from ~5700 volunteers aged 3 to 79 years from 16 collection sites stratified in five regions across Canada: Atlantic, Quebec, Ontario, Prairies, and British Columbia. The survey was designed to provide national estimates for children 3–5 years and for ages 6–11, 12–19, 20–39, 40–59, and 60–79 years for both sexes. Cycle 3 included members of the populations of the 10 provinces. Persons living in the territories, living on reserves and other aboriginal settlements in the provinces, full-time members of the Canadian Forces, the institutionalized, and persons living in certain remote areas were excluded from the survey. Altogether, these exclusions represent ~4% of the target population. The CHMS cycle 3 was approved by the Health Canada and Public Health Agency of Canada Research Ethics Board. Informed written consent was obtained from all participants older than 14 years of age. Parents or legal guardians provided written consent for younger children and the child gave assent. More detailed information on the aims of the CHMS, target population, and methodologies can be found elsewhere [38].

### 2.2. Biochemical Measurements

Fasted (≥12 h) and nonfasted blood samples were collected and processed in a MEC. Whole blood was collected in 10 mL K_2_EDTA tubes (CABD366643L, VWR International, Mississauga, ON, Canada). Serum was isolated from blood collected in 4 mL serum tubes (CABD367812L, VWR International). Whole blood and serum samples were shipped to the Nutrition Laboratory, Health Canada for measurement of HbA_1c_ (whole blood) and serum Mg, albumin, triglyceride, and glucose concentrations using the Vitros 5.1 FS clinical chemistry analyzer (Ortho Clinical Diagnostics, Mississauga, ON, Canada). Serum insulin concentration was measured using the Advia Centaur XP immunoassay analyzer (Siemens Healthcare Diagnostics, Mississauga, ON, Canada). The inter-assay (within lab precision) coefficients of variability (low–high concentration range) for HbA_1c_, Mg, albumin, triglycerides, glucose and insulin were 1.9%–3.1%, 1.7%–0.9%, 1.7%–0.9%, 1.4%–0.9%, 1.5%–1.2%, and 5.9%–4.8%, respectively. Triglycerides and insulin were only measured in fasted participants. Results from samples with degree of hemolysis exceeding the threshold value for the assay were excluded from the analyses.

### 2.3. Collection of Demographic Information

Information on age, sex, race (i.e., white, South Asian, Chinese, black, Filipino, Latin American, Arab, Southeast Asian, West Asian, Korean, Japanese, other), diabetes, and yearly household income was collected at a household interview with the participants. The interview was conducted by trained interviewers using a computer-assisted interviewing method [38].

### 2.4. Calculations

QUICKI, HOMA-IR, and McAuley’s index were calculated using the following equations: QUICKI, (log insulin (µIU·mL^−1^) + log glucose (mg·dL^−1^))^−1^; HOMA-IR, (glucose (mmol·L^−1^) × insulin (µIU·mL^−1^)) 22.5^−1^; and McAuley’s index, exp(2.63 − 0.28 ln insulin (µIU mL^−1^) − 0.31 ln triglycerides (mmol·L^−1^)). BMI was calculated from weight and height measurements.

### 2.5. Statistical Analyses

The CHMS cycle 3 sampling design yields national estimates when survey weights are applied. Bootstrap weights were used for all variance estimations to account for the complex sampling design [39]. The 16 collection sites from five regional strata restricted the statistical analyses to 11 degrees of freedom. Descriptive statistics are presented as arithmetic means and percentiles with 95% confidence intervals. A *t*-test or ANOVA followed by Tukey’s test was used for pairwise comparison of means. Association of serum Mg concentration with demographic and biochemical characteristics were assessed via linear mixed models. Age, sex, race (white or non-white), diabetes (type 1 and type 2 combined), BMI, and yearly household income were designated as fixed effects. HbA_1c_, QUICKI, HOMA-IR, McAuley’s index, and serum albumin, glucose, insulin, and triglyceride concentrations were modeled as random effects to account for the inherent variability associated with these measurements during sampling. Model 1 included the full adult sample set (fasted and nonfasted participants), and associations between Mg concentrations and age, sex, race, diabetes, BMI, household income, albumin, and HbA_1c_ were examined. Two additional models were developed using the fasted subsample. In model 2, associations with glucose, insulin, and triglycerides were investigated in addition to the variables examined in Model 1. In Model 3, associations with the indirect indices of insulin resistance QUICKI, HOMA-IR, and McAuley’s index were examined instead of the direct measures glucose, insulin, and triglycerides. The SURVEYREG procedure with backwards elimination was used to develop the final models. For the purpose of interpretation, estimates for the continuous variables were also determined after a transformation using the 5th and 95th percentiles:

(1) Y = (X−5th percentile of X) ÷ (95th percentile of X − 5th percentile of X)

Pregnant women (*n* = 19) and participants with a missing or invalid value were excluded from the analyses. The coefficients of variation for all estimates were <16.6% and considered acceptable for unrestricted release based on the CHMS sampling variability guidelines [40]. Statistical significance was set a *p* < 0.05. Statistical analyses were performed using SAS/STAT^®^ 9.3 software (SAS Institute Inc., Cary, NC, USA).

## 3. Results

**Figure 1:**
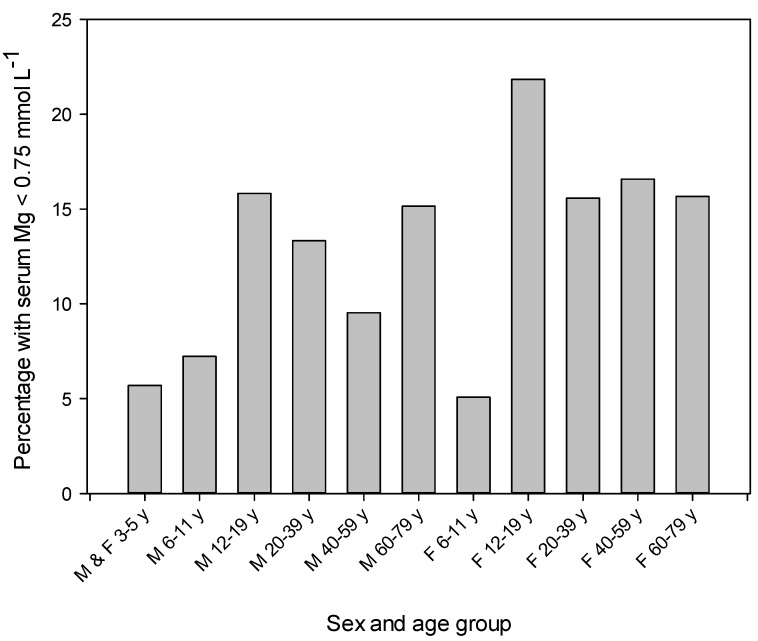
Percentage of each sex and age group with a serum Mg concentration below 0.75 mmol·L^−1^. The total number of participants (*n*) examined for each sex-age group is shown in Table 1. F, females; M, males; y, years.

Means and percentile distributions for total serum Mg concentration for 11 sex-age groups between 3 and 79 years are presented in Table 1. Distributions were symmetrical and thus arithmetic means were reported. In general, differences (*p* < 0.05) among sex and age groups were small. For ages 6–11 years, 20–39 years and 40–59 years females had lower means compared to males. Estimations for adolescents and adults at the 10th percentile were below a population-based reference interval for adults of 0.75–0.955 mmol·L^−1^ [14]. Between 9.5% and 16.6% of the adult sex-age groups had a serum Mg concentration below 0.75 mmol·L^−1^ (Figure 1). Estimates for the 25th and 95th percentiles for all sex-age groups were within the reference interval (Table 1). Boxplots show a greater number of older adults of both sexes with serum Mg concentration below the lower fence (i.e., 1.5 × interquartile range) (Figure S1).

Mean serum Mg concentration between fasted and nonfasted participants were similar (*p* ≥ 0.05) for most sex-age groups (Table S1). However, fasted males and females 6–11 years had lower means than corresponding nonfasted participants. Conversely, fasted females aged 60–79 years had a higher mean compared to non-fasting females of the same age range.

Association of serum Mg concentration with demographic factors, diabetes and measures of glycemic control and insulin resistance were examined in adults aged 20–79 years using mixed models (data for children and adolescents aged 3–19 years were excluded from these analyses). Serum albumin concentration was included in the models because of a linear relationship with serum Mg at high and low concentrations [41]. For continuous variables associations were estimated for a defined unit change and after transformation using the 5th and 95th percentiles. After transformation the estimated change in serum Mg corresponds to the change in the continuous variable from the 5th to the 95th percentile in the population. This is a better indication of the relative strengths of the associations among variables since the magnitudes of associations are compared without confounding by their scales. Estimates of the 5th and 95th percentiles for each continuous variable are presented for each model.

In Model 1, associations were estimated in fasted and nonfasted adults (Table 2). Being male was associated with higher serum Mg, whereas white race (compared to non-white) or having diabetes (type 1 or type 2) was associated with lower Mg concentrations. Age, household income, and serum albumin concentration were positively associated with serum Mg, while BMI and HbA_1c_ showed a negative association.

In Models 2 and 3, associations were examined in fasted adults. In Model 2, serum Mg was positively associated with age and household income and negatively associated with diabetes and serum glucose and insulin concentrations (Table 3). In Model 3, age and household income showed positive associations, whereas diabetes and HOMA-IR showed negative associations (Table 4).

## 4. Discussion

This study describes current (2012–2013) estimates of serum Mg concentrations in a nationally representative sample of the Canadian population for ages 3–79 years. These results are the first national estimates in Canada or the United States since the NHANES I conducted between 1971 and 1973 [14]. Results from that study showed a normal distribution for serum Mg concentration, with 95% of adults aged 18–74 years having a value between 0.75 and 0.955 mmol·L^−1^. Those estimates were considered as normative for the United States population and were used to establish a population-based reference interval. Serum Mg concentrations in the present study were also normally distributed. Substantial proportions of the adult sex-age groups (9.5%–16.6%) and adolescents aged 12–19 years (15.8%–21.8%) had a serum Mg concentration below 0.75 mmol·L^−1^, the lower cut-off of the reference interval. All estimates at the 10th percentile for adolescents and adults were also below 0.75 mmol·L^−1^. In addition, means in this study were lower than means reported in the NHANES I for comparable sex-age groups [14]. Collectively, these results suggest that present-day serum Mg concentrations in Canada are lower compared to concentrations in the United States population in the early 1970s. Since it has been suggested that a serum Mg concentration below 0.75 mmol·L^−1^ represents relatively severe Mg deficiency [11], these results raise suspicions of Mg deficiency in the Canadian population. Estimates at the 95th percentile for all sex-age groups were within the reference interval indicating the rarity of hypermagnesaemia in the population.

Similar to results from the NHANES I, only small differences in serum Mg concentrations were found among sex and age groups. Serum Mg concentrations were highest for children and showed a small increase with age from adolescents 12–19 years to older adults 60–79 years. Among demographic factors, age was the strongest predictor of serum Mg concentration in adults. The magnitudes of associations with sex, race, and household income were considerably smaller. A major finding from the NHANES I was the lower serum Mg in black Americans compared to white Americans [14]. In the present study, being white was associated with a lower serum Mg concentration than being non-white. It should be mentioned that because of the small sample sizes for all racial groups other than whites (including blacks), comparison among races in this study was restricted to the general groups of whites and non-whites.

Boxplot analyses revealed a greater number of older adults with serum Mg values below the lower fence (outliers) indicating that older adults are more prone to marked hypomagnesaemia. Possible explanations include lower dietary Mg intakes, reduced gastrointestinal Mg absorption, and increased renal Mg excretion in the elderly [42]. The higher occurrence of health conditions (e.g., diabetes) and other factors (e.g., use of hypermagnesuric diuretics) that alter Mg metabolism may also contribute [43].

Serum Mg concentrations were compared between fasted and nonfasted participants. Mean serum Mg concentrations were found to be lower for fasted children aged 6–11 years, but higher for fasted females aged 60–79 years. It is presently unclear what accounts for these differences. While the differences could be considered modest, these data offer a caution when interpreting results from a routine serum Mg test for these sex-age groups.

Inverse associations between serum Mg and diabetes and comorbidities such as poor glycemic control and insulin resistance have been well established. However, it is likely that these relationships are affected by factors that can differ among countries or change in populations over time (e.g., dietary Mg intakes, supplement and medication use). Thus, in this study associations of serum Mg concentration with diabetes and measures of glycemic regulation and insulin resistance were investigated in adults to determine the magnitude of these associations in a relatively current nationally-representative dataset. Diabetes was a strong predictor of serum Mg concentration. The estimated lower serum Mg in diabetics (ranging from −0.04 to −0.07 mmol·L^−1^ in the various models) accounts for ~20% to 34% of the spread between the lower and upper limits of the normal reference interval. These estimates are substantial and likely have clinical relevance. Serum Mg was also negatively associated with HbA_1c_ and fasting serum glucose concentration. A negative effect of diabetes on Mg status is noteworthy given the high prevalence of diabetes in Canada which is expected to rise to over 10% by the year 2020 [37].

The hyperinsulinemic euglycemic clamp is considered the “gold standard” for assessment of insulin sensitivity, but the complexity of the method limits its use in large studies [44]. In this study, associations between serum Mg and more practical surrogate indices of insulin resistance [45] were examined. Fasting insulin concentration correlates strongly with insulin resistance [46] and HOMA-IR is considered a robust tool for the evaluation of insulin resistance in large epidemiological studies [47,48]. Serum Mg showed a negative association with both fasting insulin and HOMA-IR, indicating a positive relationship between serum Mg and insulin sensitivity. Notably, these relationships were observed in models controlling for diabetes. It should be mentioned that HOMA-IR was determined to be a better predictor of serum Mg concentration compared to other proxy measures of insulin resistance such as QUICKI or McAuley’s index that were insignificant (*p* ≥ 0.05) in our selection process.

There is evidence indicating that the relationship between Mg deficiency and type 2 diabetes is bidirectional. Mg deficiency is a common manifestation in type 1 and type 2 diabetics [26,27] and may also increase the risk of diabetes and its complications [29,30,31]. The negative associations between serum Mg and measures of glycemic regulation observed in this study are in agreement with studies indicating that poor glycemic control lowers serum Mg concentrations [33,34]. However, lower serum Mg may also contribute to poorer glycemic regulation and insulin sensitivity. Studies indicate that Mg supplementation improves glucose and insulin sensitivity parameters [49,50]. A meta-analysis of randomized controlled trials showed that Mg supplementation for over four months improved fasting glucose and HOMA-IR in diabetic and non-diabetic subjects [49]. In another meta-analysis, Mg supplementation was shown to reduce fasting plasma glucose in diabetics and improve HOMA-IR and plasma glucose (in a 2 h oral glucose tolerance test) in persons at high risk of diabetes [50]. The mechanisms by which Mg deficiency may increase diabetes risk are poorly understood but may involve increased oxidative stress and inflammation [35,51,52]. It is important to mention that this study does not provide any information on the temporality of the reported associations with serum Mg given the cross-sectional design.

The main strength of this study is the CHMS cycle 3 study design, including the large sample size that is representative of the Canadian population that is ideal for estimating national serum Mg concentrations and population distributions. The use of mixed models and continuous variables (rather than dichotomizing continuous variables) is also a strength. Limitations include the absence of Mg intake data (including supplement use) for comparison with serum Mg concentrations. The different methodologies used to measure serum Mg in this study and the NHANES I (colorimetric vs. atomic absorption spectroscopy) limits, to some extent, the comparison of results between studies; however, serum Mg measurements by the colorimetric method (the most commonly used method) or atomic absorption show excellent agreement (*R* > 0.99) [53]. Also, based on the responses to the household questionnaire, many diabetics in this study could not be categorized as having type 1 or type 2 diabetes. Thus, in our analyses, diabetics included participants with type 1 or type 2 diabetes or both.

The limitations of total serum Mg concentration as a biomarker of Mg status also merit discussion. Only a small fraction (~0.3%) of total body Mg is present in the serum and fluctuations in serum proteins such as albumin can alter Mg concentrations [41]. Furthermore, serum Mg may not always accurately reflect intracellular Mg deficiency. Serum Mg concentration is tightly regulated primarily at the level of renal excretion. Bone also maintains circulating concentrations by acting as a store for Mg and supplementing the serum under conditions of deficiency. This homeostatic regulation is likely a major factor accounting for the poor association observed between serum Mg concentrations and dietary Mg intakes, particularly when intakes meet nutrient requirements [54,55]. Despite these limitations, low serum Mg is usually indicative of Mg deficiency. Serum Mg is decreased in a dose-dependent manner in animal models of dietary Mg deficiency, demonstrating that low Mg intakes (Mg deficiency) reduce serum Mg concentration [56,57]. Human studies have shown that serum Mg is responsive to long-term changes in dietary Mg intakes [58] and increases with Mg supplementation [13,59,60]. A meta-analysis of randomized controlled trials showed that serum Mg concentrations increase in a dose- and time-dependent manner with oral Mg supplementation, and the response is greater when baseline circulating Mg concentrations are lower, suggesting that serum Mg provides useful information about underlying Mg status [13]. It has been suggested that a serum Mg value below 0.75 mmol·L^−1^ is a useful measure of relatively severe Mg deficiency, but Mg deficiency cannot be excluded for persons with a value between 0.75 and 0.85 mmol·L^−1^ [11,12].

## 5. Conclusions

This study reports serum Mg concentrations for 11 sex-age groups between 3 and 79 years in the Canadian population. Between 9.5% and 16.6% of the adult sex-age groups were hypomagnesaemic (serum Mg < 0.75 mmol·L^−1^) in relation to a population-based reference interval [14]. There is a need to establish an evidence-based reference interval (or at minimum a lower cut-off value) for health that will allow more accurate assessment of the prevalence of Mg deficiency in Canada and related health risk based on results from this study [61]. Among demographic factors, age was the strongest predictor of serum Mg concentration. Serum Mg concentration was negatively associated with diabetes, BMI, serum glucose, serum insulin, HbA_1c_, and HOMA-IR. These results are consistent with a growing body of evidence indicating a negative effect of diabetes, poor glycemic control, and insulin resistance on Mg status.

## Figures and Tables

**Table 1:** Means and distributions of serum magnesium concentrations by sex and age in the Canadian population.

Sex and Age	*n*	Serum Magnesium		Distribution of Serum Magnesium Concentrations													
				5th		10th		25th		50th		75th		90th		95th	
		Arithmetic Mean ^1^	95% CI	Estimate	95% CI	Estimate	95% CI	Estimate	95% CI	Estimate	95% CI	Estimate	95% CI	Estimate	95% CI	Estimate	95% CI
		mmol·L^−1^															
All ^2^																	
3–5 years	505	0.83	0.82, 0.84	0.74	0.72, 0.76	0.76	0.74, 0.77	0.78	0.77, 0.80	0.82	0.80, 0.84	0.86	0.84, 0.88	0.90	0.87, 0.93	0.92	0.90, 0.95
Male ^3^																	
6–11 years	493	0.83 ^a,^*	0.82, 0.84	0.73	0.71, 0.76	0.76	0.73, 0.78	0.79	0.78, 0.81	0.83	0.82, 0.84	0.86	0.85, 0.88	0.88	0.86, 0.91	0.91	0.88, 0.94
12–19 years	490	0.80 ^c^	0.78, 0.81	0.71	0.68, 0.74	0.73	0.70, 0.75	0.76	0.72, 0.79	0.79	0.77, 0.81	0.83	0.80, 0.85	0.87	0.84, 0.89	0.88	0.87, 0.90
20–39 years	510	0.81 ^b,c,^*	0.80, 0.82	0.69	0.64, 0.74	0.73	0.70, 0.76	0.77	0.74, 0.79	0.80	0.79, 0.82	0.84	0.83, 0.86	0.87	0.86, 0.89	0.90	0.87, 0.92
40–59 years	538	0.82 ^a,b,^*	0.81, 0.83	0.71	0.68, 0.73	0.74	0.71, 0.77	0.78	0.76, 0.80	0.82	0.80, 0.84	0.86	0.84, 0.88	0.90	0.88, 0.92	0.91	0.88, 0.94
60–79 years	509	0.81 ^b^	0.81, 0.82	0.69	0.67, 0.71	0.72	0.71, 0.74	0.77	0.75, 0.79	0.82	0.80, 0.84	0.86	0.84, 0.87	0.89	0.88, 0.91	0.91	0.89, 0.93
Female ^3^																	
6–11 years	455	0.82 ^a^	0.81, 0.83	0.74	0.72, 0.76	0.75	0.73, 0.78	0.78	0.77, 0.80	0.82	0.80, 0.84	0.85	0.83, 0.86	0.88	0.86, 0.89	0.89	0.88, 0.90
12–19 years	486	0.79 ^d^	0.78, 0.80	0.70	0.68, 0.72	0.72	0.70, 0.73	0.75	0.73, 0.77	0.78	0.77, 0.80	0.82	0.80, 0.83	0.86	0.84, 0.87	0.88	0.86, 0.90
20–39 years	511	0.80 ^c,d^	0.79, 0.80	0.71	0.70, 0.72	0.73	0.71, 0.75	0.76	0.74, 0.78	0.79	0.77, 0.80	0.82	0.80, 0.84	0.85	0.82, 0.88	0.87	0.85, 0.89
40–59 years	532	0.81 ^b,c^	0.80, 0.82	0.69	0.64, 0.74	0.72	0.69, 0.76	0.76	0.74, 0.78	0.81	0.79, 0.82	0.85	0.83, 0.87	0.88	0.86, 0.89	0.89	0.87, 0.92
60–79 years	532	0.82 ^a,b^	0.81, 0.83	0.67	0.65, 0.69	0.71	0.69, 0.73	0.77	0.75, 0.79	0.82	0.80, 0.83	0.86	0.84, 0.88	0.90	0.88, 0.92	0.93	0.90, 0.97

^1^ Values in a column and within a sex group without a common superscript letter differ, *p* < 0.05. * Different compared to females in the same age group, *p* < 0.05; ^2^ Includes nonfasted males and females; ^3^ Includes fasted and nonfasted participants.

**Table 2:** Association of serum Mg with demographic factors, diabetes and biochemical measures ^1^.

IV	Estimate (95% CI) ^2,3^	Estimate (95% CI) ^3,4^	*p*	Distribution of Continuous IV	
	mmol·L^−1^			5th (95% CI )	95th (95% CI )
Male ^5^	0.01 (0.00, 0.01)	-	<0.01	-	-
White race ^6^	−0.01 (−0.02, −0.00)	-	<0.001	-	-
Diabetes ^7^	−0.04 (−0.05, −0.02)	-	<0.001	-	-
Age, year	0.01 (0.01, 0.01)	0.05 (0.04, 0.05)	<0.001	21.1 (18.3, 23.9)	71.7 (70.1, 73.3)
BMI, kg·m^−2^	−0.002 (−0.002, −0.001)	−0.03 (−0.03, −0.02)	<0.001	19.7 (19.1, 20.3)	36.3 (34.7, 38.0)
Household income, K	0.0005 (0.0001, 0.0008)	0.01 (0.00, 0.01)	<0.01	15.0 (11.5, 18.5)	196.7 (166.2, 227.2)
Serum albumin, g·L^−1^	0.002 (0.001, 0.003)	0.02 (0.01, 0.03)	<0.001	37.8 (36.3, 39.3)	48.9 (47.4, 50.5)
HbA_1c_, %	−0.01 (−0.02, −0.01 )	−0.02 (−0.03, −0.02)	<0.001	4.8 (4.6, 5.0)	6.5 (6.1, 6.9)

^1^ Results from fasted and nonfasted adults aged 20–79 years, *n* = 2838 (Model 1). Sex, race, diabetes, age, BMI, household income, serum albumin concentration and HbA_1c_ were tested in the model. All variables were statistically significant (*p* < 0.05) and retained in the final model. HbA_1c_, glycated hemoglobin; IV, independent variables; Mg, magnesium; ^2^ Changes in serum Mg concentrations associated with being male (compared to female), white race (compared to non-white race), diabetes, a 10 years increment in age, a 1 kg·m^−2^ increment in BMI, a $10 K increment in yearly household income, a 1 g·L^−1^ increment in serum albumin concentration and a 1% increment in HbA_1c_; ^3^ Estimates are adjusted for all IV in the model; ^4^ Continuous variables were transformed using the 5th and 95th percentiles prior to analysis; ^5^ Males, *n* = 1438; ^6^ Whites, *n* = 2331; ^7^ Diabetics, *n* = 217.

**Table 3:** Association of serum Mg with demographic factors, diabetes and serum glucose and insulin concentrations ^1^.

IV	Estimate (95% CI) ^2,3^	Estimate (95% CI) ^3,4^	*p*	Distribution of Continuous IV	
	mmol·L^−1^			5th (95% CI )	95th (95% CI )
Diabetes ^5^	−0.06 (−0.07, −0.04)	-	<0.001	-	-
Age, year	0.01 (0.01, 0.01)	0.04 (0.03, 0.05)	<0.001	20.6 (17.9, 23.3)	70.8 (69.1, 72.4)
Household income, K	0.0008 (0.0004, 0.0012)	0.02 (0.01, 0.02)	<0.001	14.5 (10.3, 18.7)	199.0 (162.5, 235.6)
Serum glucose, mmol·L^−1^	−0.01 (−0.01, −0.00)	−0.01 (−0.02, −0.01)	<0.001	4.3 (4.1, 4.4)	6.6 (5.9, 7.4)
Serum insulin, pmol·L^−1^	−0.00008 (−0.00013, −0.00004)	−0.01 (−0.02, −0.00)	<0.001	25.1 (21.9, 28.2)	180.1 (156.7, 203.4)

^1^ Results from fasted adults aged 20–79 years, *n* = 1621 (Model 2). Sex, race, diabetes, age, BMI, household income, serum albumin concentration, HbA_1c_, serum glucose concentration, serum insulin concentration and serum triglyceride concentration were tested in the model. Statistically significant (*p* < 0.05) variables were selected by backwards elimination and retained in the final model. IV, independent variables; Mg, magnesium; ^2^ Changes in serum Mg concentrations associated with diabetes, a 10 years increment in age, a $10 K increment in yearly household income, a 1 mmol·L^−1^ increment in serum glucose concentration and a 1 pmol·L^−1^ increment in serum insulin concentration; ^3^ Estimates are adjusted for all IV in the model; ^4^ Continuous variables were transformed using the 5th and 95th percentiles prior to analysis; ^5^ Diabetics, *n* = 99.

**Table 4:** Association of serum Mg with demographic factors, diabetes and HOMA-IR ^1^.

IV	Estimate (95% CI) ^2,3^	Estimate (95% CI) ^3,4^	*p*	Distribution of Continuous IV	
	mmol·L^−1^			5th (95% CI )	95th (95% CI )
Diabetes ^5^	−0.07 (−0.08, −0.06)	-	<0.001	-	-
Age, year	0.01 (0.01, 0.01)	0.04 (0.03, 0.05)	<0.001	20.6 (18.0, 23.3)	70.8 (69.1, 72.5)
Household income, K	0.0008 (0.0004, 0.0012)	0.02 (0.01, 0.02)	<0.001	14.5 (10.3, 18.7)	199.0 (162.0, 236.0)
HOMA-IR	−0.003 (−0.004, −0.002)	−0.02 (−0.02, −0.01)	<0.001	0.80 (0.67, 0.93)	6.99 (6.34, 7.65)

^1^ Results from fasted adults aged 20–79 years, *n* = 1621 (Model 3). Sex, race, diabetes, age, BMI, household income, serum albumin concentration, HbA_1c_, serum triglyceride concentration and HOMA-IR were tested in the model. Statistically significant (*p* < 0.05) variables were selected by backwards elimination and retained in the final model. HOMA-IR, homeostatic model assessment of insulin resistance; IV, independent variables; Mg, magnesium; ^2^ Changes in serum Mg concentrations associated with diabetes, a 10 years increment in age, a $10 K increment in yearly household income and an increment of 1 for HOMA-IR; ^3^ Estimates are adjusted for all IV in the model; ^4^ Continuous variables were transformed using the 5th and 95th percentiles prior to analysis; ^5^ Diabetics, *n* = 99.

## Supplementary Materials

The following are available online at http://www.mdpi.com/2072-6643/9/3/296/s1, Figure S1: Boxplots of serum Mg concentrations by sex-age group, Table S1: Means and distributions of serum magnesium concentrations by sex and age in fasted and nonfasted Canadians.
