# Supplementary material for: Serum Magnesium Concentrations in the Canadian Population and Associations with Diabetes, Glycemic Regulation, and Insulin Resistance
Source: Nutrients. 2017 Mar 17;9(3):296. doi: 10.3390/nu9030296 (PMC5372959; doi:10.3390/nu9030296)
Supplement: Supplementary file 1 [file nutrients-09-00296-s001.pdf]

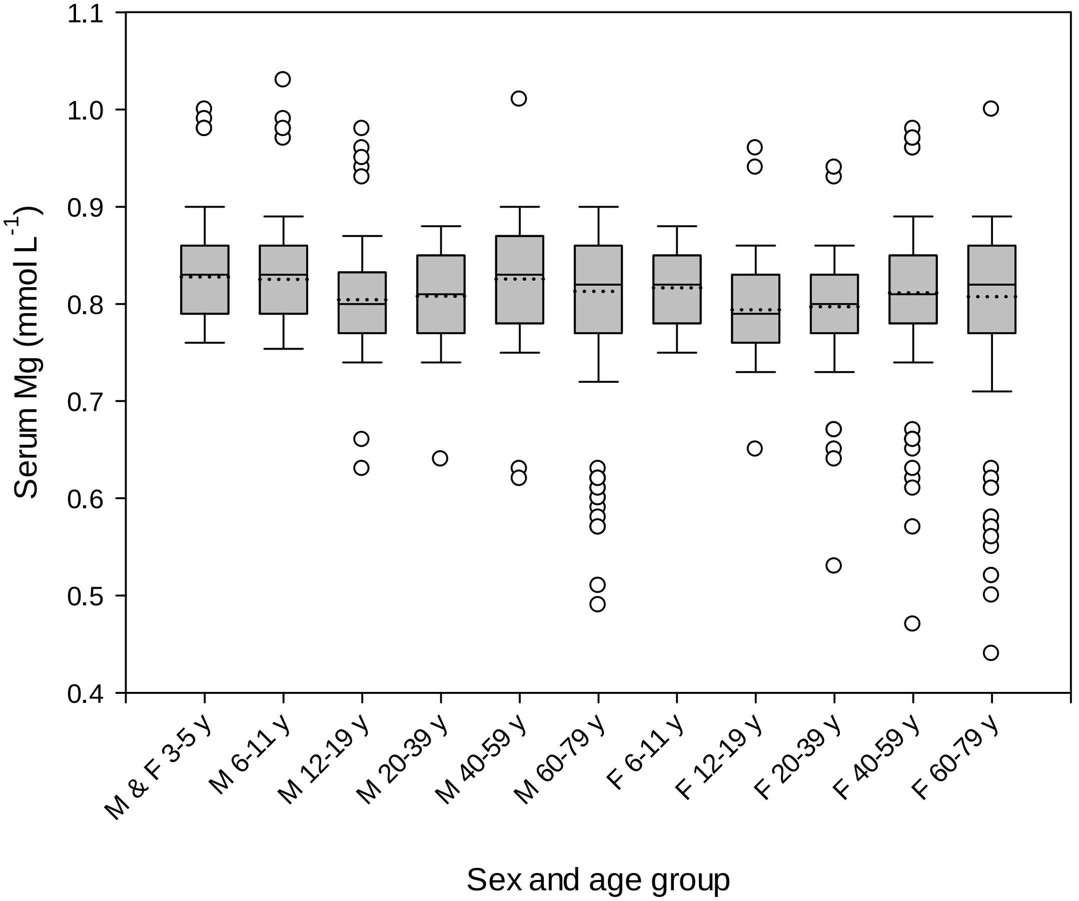

Figure S1. Boxplots of serum Mg concentrations by sex-age group. Open circles represent outliers (values greater or less than 1.5 × interquartile range). The median (solid line) and mean (dashed line) for each group is shown within the box. Whiskers represent the 10th and the 90th percentiles. The number of participants in each group is the same as shown in Table 1. F, females; M, males.

Table S1. Means and distributions of serum magnesium concentrations by sex and age in fasted and nonfasted Canadians.

Sex and age	n	Serum magnesium		Distribution of serum magnesium concentrations													
				5th		10th		25th		50th		75th		90th		95th	
		Arithmetic mean[1]	95% CI	Estimate	95% CI	Estimate	95% CI	Estimate	95% CI	Estimate	95% CI	Estimate	95% CI	Estimate	95% CI	Estimate	95% CI
		mmol L^{-1}															
		Fasted															
Male																	
6–11 y	242	$0.82^{*,\#}$	0.81, 0.82	0.73	0.72, 0.75	0.75	0.73, 0.77	0.78	0.76, 0.80	0.82	0.80, 0.83	0.84	0.83, 0.86	0.87	0.85, 0.89	0.89	0.87, 0.90
12–19 y	244	0.80	0.79, 0.82	0.73	0.71, 0.75	0.74	0.72, 0.76	0.76	0.74, 0.79	0.79	0.77, 0.82	0.83	0.81, 0.85	0.87	0.84, 0.89	0.88	0.86, 0.90
20–39 y	270	0.81	0.80, 0.82	0.71	0.65, 0.78	0.74	0.72, 0.77	0.78	0.76, 0.80	0.80	0.79, 0.82	0.84	0.82, 0.85	0.87	0.84, 0.89	0.89	0.85, 0.94
40–59 y	294	0.81	0.79, 0.84	0.70	0.64, 0.76	0.72	0.66, 0.78	0.77	0.74, 0.81	0.81	0.78, 0.84	0.85	0.81, 0.88	0.89	0.86, 0.92	0.90	0.87, 0.94
60–79 y	264	0.82	0.81, 0.83	0.71	0.66, 0.77	0.74	0.72, 0.76	0.77	0.75, 0.79	0.82	0.80, 0.84	0.86	0.85, 0.88	0.90	0.88, 0.92	0.91	0.90, 0.93
Female																	
6–11 y	203	$0.80^{b,\#}$	0.80, 0.81	0.73	0.71, 0.74	0.74	0.73, 0.76	0.77	0.75, 0.79	0.80	0.78, 0.82	0.83	0.82, 0.85	0.86	0.84, 0.87	0.87	0.85, 0.88
12–19 y	239	0.79^{b}	0.78, 0.80	0.70	0.68, 0.73	0.73	0.70, 0.76	0.75	0.73, 0.77	0.78	0.77, 0.80	0.83	0.81, 0.84	0.86	0.84, 0.87	0.87	0.85, 0.89
20–39 y	269	0.80^{b}	0.78, 0.81	0.70	0.66, 0.75	0.73	0.70, 0.76	0.76	0.72, 0.79	0.80	0.78, 0.82	0.83	0.80, 0.86	0.85	0.82, 0.89	0.87	0.85, 0.90
40–59 y	249	0.80^{b}	0.79, 0.82	0.70	0.67, 0.73	0.73	0.70, 0.76	0.75	0.73, 0.77	0.80	0.77, 0.83	0.84	0.82, 0.86	0.87	0.84, 0.91	0.92	0.86, 0.97
60–79 y	278	$0.83^{a,\#}$	0.81, 0.85	0.69	0.66, 0.73	0.73	0.70, 0.77	0.79	0.76, 0.82	0.83	0.81, 0.85	0.88	0.86, 0.91	0.91	0.89, 0.93	0.92	0.88, 0.97
		Nonfasted															
Male																	
6–11 y	251	0.85^{a}	0.83, 0.86	0.75	0.72, 0.79	0.77	0.74, 0.80	0.81	0.79, 0.82	0.84	0.83, 0.85	0.87	0.85, 0.89	0.90	0.87, 0.93	0.93	0.88, 0.97
12–19 y	246	0.80^{c}	0.78, 0.81	0.69	0.64, 0.74	0.72	0.69, 0.75	0.75	0.72, 0.79	0.79	0.77, 0.81	0.83	0.80, 0.85	0.87	0.84, 0.90	0.89	0.86, 0.91
20–39 y	240	0.80^{c}	0.79, 0.81	0.68	0.65, 0.72	0.71	0.67, 0.75	0.76	0.73, 0.79	0.81	0.79, 0.82	0.83	0.81, 0.85	0.88	0.85, 0.90	0.89	0.86, 0.92
40–59 y	244	$0.83^{ab,*}$	0.82, 0.84	0.72	0.68, 0.75	0.75	0.71, 0.78	0.79	0.77, 0.81	0.83	0.80, 0.85	0.86	0.84, 0.89	0.91	0.88, 0.93	0.93	0.90, 0.96
60–79 y	245	0.81^{bc}	0.80, 0.82	0.69	0.67, 0.70	0.71	0.67, 0.74	0.76	0.73, 0.79	0.81	0.79, 0.83	0.85	0.84, 0.87	0.89	0.87, 0.91	0.91	0.87, 0.94
Female																	
6–11 y	252	0.83^{a}	0.82, 0.84	0.76	0.73, 0.78	0.77	0.75, 0.79	0.79	0.77, 0.81	0.83	0.82, 0.85	0.86	0.85, 0.88	0.88	0.87, 0.90	0.89	0.88, 0.91
12–19 y	247	0.79^{c}	0.78, 0.80	0.70	0.67, 0.73	0.71	0.70, 0.73	0.74	0.71, 0.77	0.78	0.77, 0.80	0.81	0.80, 0.83	0.85	0.83, 0.87	0.88	0.86, 0.90
20–39 y	242	0.79^{bc}	0.78, 0.81	0.71	0.69, 0.73	0.72	0.69, 0.75	0.76	0.74, 0.79	0.78	0.77, 0.80	0.82	0.79, 0.85	0.86	0.84, 0.88	0.87	0.84, 0.91
40–59 y	283	0.81^{b}	0.80, 0.82	0.65	0.52, 0.78	0.73	0.68, 0.78	0.77	0.75, 0.79	0.81	0.79, 0.83	0.86	0.84, 0.87	0.87	0.86, 0.89	0.89	0.87, 0.90
60–79 y	254	0.81^{bc}	0.79, 0.81	0.66	0.63, 0.68	0.70	0.66, 0.74	0.75	0.73, 0.77	0.81	0.79, 0.82	0.85	0.84, 0.87	0.89	0.84, 0.94	0.94	0.89, 1.00

[1] For fasted or nonfasted participants, values in a column and within a sex group without a common superscript letter differ, $p < 0.05$. * Different compared to females in the same age group and fasted or nonfasted state, $p < 0.05$. # Different compared to nonfasted participants in the same sex-age group, $p < 0.05$.
